# Real-World Applications of Imipenem-Cilastatin-Relebactam: Insights From a Multicenter Observational Cohort Study

**DOI:** 10.1093/ofid/ofaf112

**Published:** 2025-02-26

**Authors:** Kaylee E Caniff, Nicholas Rebold, Xhilda Xhemali, Nikki Tran, Taryn A Eubank, Kevin W Garey, Yi Guo, Mei Chang, Katie E Barber, Tamara Krekel, Mark Biagi, Wesley D Kufel, Amy Carr, Jillian Hayes, Travis J Carlson, Jeremy Frens, Veena Venugopalan, Kristen Lucas, Ashlan J Kunz Coyne, James Sanders, Elisabeth Chandler, Rosanna Li, Kayla Antosz, Julie Ann Justo, Russell Benefield, W Justin Moore, Jennifer Ross, Jenna Adams, Fritzie Albarillo, Sylvia Stefanos, Athena L V Hobbs, Nicholas Mercuro, Brian Raux, Kristen Zeitler, Michael J Rybak

**Affiliations:** Anti-Infective Research Laboratory, Eugene Applebaum College of Pharmacy and Health Sciences, Wayne State University, Detroit, Michigan, USA; Anti-Infective Research Laboratory, Eugene Applebaum College of Pharmacy and Health Sciences, Wayne State University, Detroit, Michigan, USA; Department of Clinical & Administrative Pharmacy Sciences, Howard University College of Pharmacy, Washington, DC, USA; Department of Pharmacy, Cleveland Clinic, Cleveland, Ohio, USA; Department of Pharmacy, The Ohio State University Wexler Medical Center, Columbus, Ohio, USA; Department of Pharmacy Practice and Translational Research, University of Houston College of Pharmacy, Houston, Texas, USA; Department of Pharmacy Practice and Translational Research, University of Houston College of Pharmacy, Houston, Texas, USA; Department of Pharmacy, Montefiore Medical Center, The Bronx, New York, USA; Department of Pharmacy, Montefiore Medical Center, The Bronx, New York, USA; Department of Pharmacy, University of Mississippi Medical Center, Jackson, Mississippi, USA; Department of Pharmacy, Barnes-Jewish Hospital, St Louis, Missouri, USA; Department of Pharmacy, UW Health SwedishAmerican Hospital, Rockford, Illinois, USA; Department of Pharmacy, State University of New York Upstate Medical University, Syracuse, New York, USA; Department of Pharmacy Practice, Binghamton University School of Pharmacy and Pharmaceutical Sciences, Binghamton, New York, USA; Department of Pharmacy, AdventHealth Orlando, Orlando, Florida, USA; Department of Pharmacy, AdventHealth Orlando, Orlando, Florida, USA; Department of Pharmacy, Cone Health, Greensboro, North Carolina, USA; Department of Clinical Sciences, High Point University Fred Wilson School of Pharmacy, High Point, North Carolina, USA; Department of Clinical Sciences, High Point University Fred Wilson School of Pharmacy, High Point, North Carolina, USA; Department of Pharmacotherapy & Translational Research, University of Florida College of Pharmacy, Gainesville, Gainesville, Florida, USA; Department of Pharmacy Practice and Science, University of Kentucky College of Pharmacy, Lexington, Kentucky, USA; Department of Pharmacy Practice and Science, University of Kentucky College of Pharmacy, Lexington, Kentucky, USA; Department of Pharmacy, UT Southwestern Medical Center, Dallas, Texas, USA; Department of Pharmacy, Lee Health, Fort Myers, Florida, USA; Department of Pharmacy, Maimonides Medical Center, Brooklyn, New York, USA; Department of Pharmacy, Prisma Health Richland Hospital, Columbia, South Carolina, USA; Clinical Pharmacy and Outcomes Sciences, University of South Carolina College of Pharmacy, Columbia, South Carolina, USA; Department of Pharmacy, Prisma Health Richland Hospital, Columbia, South Carolina, USA; Clinical Pharmacy and Outcomes Sciences, University of South Carolina College of Pharmacy, Columbia, South Carolina, USA; Department of Pharmacy, University of Utah Health, Salt Lake City, Utah, USA; Department of Pharmacy, Northwestern Memorial Hospital, Chicago, Illinois, USA; Department of Pharmacy, M Health Fairview University of Minnesota Medical Center, Minneapolis, Minnesota, USA; Department of Pharmacy, Loyola University Medical Center, Maywood, Illinois, USA; Department of Medicine, Loyola University Medical Center, Maywood, Illinois, USA; Department of Pharmacy, Methodist Le Bonheur Healthcare, University Hospital, Memphis, Tennessee, USA; Department of Pharmacy, Methodist Le Bonheur Healthcare, University Hospital, Memphis, Tennessee, USA; Department of Pharmacy, Beth Israel Deaconess Medical Center, Boston, Massachusetts, USA; Department of Pharmacy, Medical University of South Carolina, Charleston, South Carolina, USA; Department of Pharmacy, Tampa General Hospital, Tampa, Florida, USA; Anti-Infective Research Laboratory, Eugene Applebaum College of Pharmacy and Health Sciences, Wayne State University, Detroit, Michigan, USA; Department of Pharmacy, Detroit Receiving Hospital, Detroit, Michigan, USA

**Keywords:** carbapenem-resistant, gram-negative, imipenem-cilastatin-relebactam, multidrug-resistant, *Pseudomonas aeruginosa*

## Abstract

**Background:**

Multidrug-resistant (MDR) gram-negative infections are a substantial threat to patients and public health. Imipenem-cilastatin-relebactam (IMI/REL) is a β-lactam/β-lactamase inhibitor with expanded activity against MDR *Pseudomonas aeruginosa* and carbapenem-resistant Enterobacterales. This study aims to describe the patient characteristics, prescribing patterns, and clinical outcomes associated with IMI/REL.

**Methods:**

This was a retrospective, multicenter, observational study of patients ≥18 years old who received IMI/REL for ≥48 hours for a suspected or confirmed gram-negative infection. The primary outcome was clinical success, defined as improvement or resolution of infection-related signs or symptoms while receiving IMI/REL and the absence of 30-day microbiologic failure. Multivariable logistic regression analysis was performed to identify independent predictors of clinical success.

**Results:**

The study included 151 patients from 24 US medical centers. IMI/REL was predominantly prescribed for lower respiratory tract infections, accounting for 52.3% of cases. Most patients were infected with a carbapenem-nonsusceptible pathogen (85.4%); *P aeruginosa* was frequently targeted (72.2%). Clinical success was achieved in 70.2% of patients. Heart failure, receipt of antibiotics within the past 90 days, intensive care unit admission at time of index culture collection, and isolation of difficult-to-treat resistant *P aeruginosa* were independently associated with a reduced odds of clinical success. Adverse events were reported in 6.0% of patients, leading to discontinuation of IMI/REL in 3 instances.

**Conclusions:**

This study provides a comprehensive analysis of the real-world effectiveness and safety of IMI/REL. Comparative studies and investigations of specific subgroups will further enhance our understanding of IMI/REL in treating MDR infections.

## Teaser

This real-world, multicenter study examined patient characteristics, microbiologic data, prescribing patterns, and clinical outcomes associated with imipenem-cilastatin-relebactam (IMI/REL). Predominantly used to target *Pseudomonas aeruginosa*, IMI/REL demonstrated a favorable safety and efficacy profile in a cohort with significant comorbid conditions and illness acuity.

Antimicrobial resistance is one of the most substantial public health threats of modern times, contributing to substantial morbidity, mortality, and healthcare costs [1]. The increasing prevalence of resistant gram-negative pathogens, such as carbapenem-resistant Enterobacterales (CRE) and multidrug-resistant (MDR) *Pseudomonas aeruginosa*, is of particular concern [2]. The Centers for Disease Control and Prevention has assigned the highest threat level to CRE and labeled MDR *P aeruginosa* as a serious threat [3]. Polymyxins and aminoglycosides were historically used to combat these infections; however, their suboptimal pharmacokinetics and unfavorable safety profiles present challenges in a population that commonly has comorbid conditions and acute organ dysfunction [4, 5]. Moreover, the poor clinical outcomes associated with these infections necessitate investigation of novel treatment strategies and new antibiotics to overcome resistance [6–8]. Encouragingly, novel β-lactam/β-lactamase inhibitor agents with activity against MDR gram-negative pathogens have increasingly been introduced into clinical practice [9–12].

Imipenem-cilastatin-relebactam (IMI/REL) is a β-lactam/β-lactamase inhibitor combination that includes a carbapenem, a renal dehydropeptidase I inhibitor, and a dual-Ambler class A/C β-lactamase inhibitor. Approved in 2019, this antimicrobial is labeled for use in complicated urinary tract or intra-abdominal infections, hospital-acquired pneumonia, and ventilator-associated pneumonia [13]. Relebactam restores the activity of imipenem against resistant organisms by inhibiting several β-lactamases, including Ambler class A β-lactamases (eg, *Klebsiella pneumoniae* carbapenemase), extended-spectrum β-lactamases, and AmpC β-lactamases. Furthermore, IMI/REL is likely unaffected by efflux pump– or OprD porin channel–mediated resistance in *P aeruginosa*, which commonly affects other carbapenems [14]. In the RESTORE-IMI 1 and RESTORE-IMI 2 clinical trials, IMI/REL was efficacious and well tolerated in patients with complicated urinary tract or intra-abdominal infection, hospital-acquired pneumonia, and ventilator-associated pneumonia, demonstrating noninferiority to comparators [15, 16].

Prospective randomized controlled trials (RCTs) are considered the reference standard for scientific evidence, but their generalizability to clinical practice can be limited, particularly in the context of MDR gram-negative infections. Patients with these infections frequently have substantial comorbid conditions and highly severe illness, often making them ineligible for RCTs evaluating novel antimicrobials [7]. Furthermore, RCTs are unable to enroll a large sample of patients with specific resistance patterns across diverse infection sources. Alternatively, retrospective observational studies offer valuable insights into practice patterns and clinical outcomes in real-world settings. In the current study, we aimed to describe clinical experience with IMI/REL for treating gram-negative infections in a practical, real-world context.

## METHODS

### Patient Inclusion and Data Collection

This was a retrospective, observational, cohort study of hospitalized patients who were treated with IMI/REL from 1 July 2019 to 31 July 2024. To be eligible for inclusion, adult patients (≥18 years) must have received ≥48 hours of IMI/REL for the treatment of a suspected or confirmed infection caused by a gram-negative pathogen. Patients were excluded if they were pregnant, nursing, or imprisoned. Subsequent IMI/REL courses were excluded unless separated by ≥60 days from the index infection.

Study data were manually extracted from the electronic medical record and managed using the Research Electronic Data Capture (REDCap) tool hosted at Wayne State University [17]. Extracted data included baseline demographics, medical history and comorbid conditions, risk factors for acquisition of MDR bacteria (eg, prior antibiotic and healthcare exposures within the previous 90 days), clinical laboratory data, microbiologic data (including organism identification and susceptibilities as available), infection source, diagnostic and therapeutic procedures, infectious diseases consultation, surgical consultation, and treatment-related parameters, including dosage and duration of all antimicrobial therapy for the index infection.

### Patient Consent Statement

This study was reviewed and approved by the Wayne State University Institutional Review Board as well as the institutional review boards at all participating centers. Patient consent was waived given the retrospective study design. Procedures followed were in accordance with the ethical standards of the Helsinki Declaration of 1964 and its later amendments and of the World Medical Association.

### Outcomes and Definitions

The primary outcome was clinical success, defined as improvement or resolution of infection-related signs or symptoms while receiving IMI/REL and the absence of microbiologic failure while receiving therapy and within 30 days of the last IMI/REL dose. The improvement of infection-related signs and symptoms was assessed during the record review. This was defined as an improvement or resolution in infection-related white blood cell count and abnormal temperature, without the need to change antibiotic therapy from IMI/REL to an alternative agent due to lack of infection-specific symptom resolution as documented by the treating provider.

Secondary outcomes included 30-day all-cause mortality after the first IMI/REL dose, 30-day microbiologic failure, 30-day infection recurrence, 30-day infection-related readmission, and length of hospital stay and adverse effects attributed to IMI/REL. Microbiologic failure was defined as the isolation of the causative pathogen during IMI/REL therapy or within 30 days from IMI/REL discontinuation; patients with resolution of clinical signs/symptoms of infection without follow-up cultures were considered to have successfully eradicated the causative organism. Infection recurrence was defined as microbiologic failure with concomitant signs/symptoms of infection within 30 days of IMI/REL discontinuation. Length of hospital stay was defined as the number of days from hospital admission to discharge. Infection-related readmission was defined as a readmission attributed to the index infection and/or an infection recurrence within 30-days of IMI/REL discontinuation. Directed therapy was defined as continuation of IMI/REL for >24 hours following culture finalization.

Severity of illness was quantified using the Acute Physiology and Chronic Health II score [18] and the comorbidity burden, using the Charlson Comorbidity Index [19]. Microbiologic susceptibility was defined using Clinical and Laboratory Standards Institute (CLSI) M100 interpretative breakpoints [20]. The targeted pathogen refers to the organism(s) identified in the index culture for which IMI/REL was used as the treatment. CRE was defined as Enterobacterales that was resistant to ≥1 carbapenems using CLSI M100 standards or demonstrated production of a carbapenemase. *P aeruginosa* resistance was defined as MDR if the isolate was nonsusceptible (intermediate or resistant) to ≥1 agent in ≥3 antimicrobial categories and as difficult-to-treat resistance (DTR) if the isolate was nonsusceptible to cefepime, ceftazidime, piperacillin-tazobactam, aztreonam, meropenem, imipenem-cilastatin, levofloxacin, and ciprofloxacin [21, 22].

Creatinine clearance (CrCl) was estimated using the Cockcroft-Gault equation on the day of IMI/REL initiation. The appropriateness of dosing was assessed by comparing the patient's CrCl with the dosing recommendations provided in the IMI/REL package insert. A patient was considered to be underdosed if prescribed a dose or frequency less than that recommended for the calculated CrCl [23]. Immunosuppression was defined as having ≥1 of the following: neutropenia (absolute neutrophil count or white blood cell count <500/μL); CD4 cell count < 200/μL [3] or AIDS-defining illness; functional or surgical splenectomy; solid organ transplant, bone marrow transplant or cytotoxic chemotherapy receipt in the past 90 days; or high-dose corticosteroid receipt (>200 mg hydrocortisone or equivalent for ≥2 weeks).

### Statistical Analysis

Descriptive analyses were performed for patient demographics, comorbid conditions, clinical characteristics, IMI/REL prescribing information, and outcomes. Count and frequency were used to describe categorical variables. The normality of continuous variables was assessed using the Shapiro-Wilk test and by plot inspection. Normally distributed continuous variables were reported as means (with standard deviation) and nonnormally distributed continuous variables as medians (with interquartile range [IQR]).

Multivariable logistic regression was performed to explore independent predictors of clinical success. Bivariate analysis was initially conducted to compare patients who experienced clinical success with those who did not. For categorical variables, χ2 tests or Fisher exact tests (for expected n < 5) were used; for continuous variables, Student *t* or Mann-Whitney *U* tests. Variables with a *P* value <.1 in the bivariate analysis, present in >10% of the overall cohort, and deemed to be clinically relevant were considered for entry into the logistic regression model. A backward stepwise approach was used to create the most parsimonious model. The variance of inflation factor was used to assess multicollinearity of covariates in the model, with an acceptable level considered to be <5. Model fit was evaluated with the Hosmer-Lemeshow goodness-of-fit test. All analyses were performed using SPSS software, version 29.0 (IBM).

## RESULTS

There were 151 cases included from 24 participating US medical centers across 15 states. Baseline demographics and patient characteristics are provided in Table 1. The median age (IQR) of the cohort was 60 (42–71) years, 54.3% were male, and the patients were predominantly non-Hispanic white (68.2%). There was a high burden of comorbid conditions, with a median (IQR) Charlson Comorbidity Index of 4 (2–6). Common comorbid conditions included diabetes mellitus (39.1%), heart failure (28.5%), and chronic kidney disease (25.8%). Notably, 15.2% of patients had an admission associated with a diagnosis of coronavirus disease 2019 (COVID-19), and 19.2% were considered immunosuppressed. Previous exposures to antimicrobials and healthcare contact were common; 74.2% and 71.5%, respectively, had received antimicrobials for ≥24 hours and had been hospitalized for ≥48 hours within the previous 90 days.

**Table 1.** Patient Characteristics

Characteristic	Patients, No. (%)^a^ (n = 151)
Age, median (IQR), y	60 (42–71)
Male sex	82 (54.3)
Race	
Non-Hispanic white	103 (68.2)
Black/African American	33 (21.9)
Hispanic	8 (5.3)
Asian	5 (3.3)
Other/unknown	2 (1.3)
BMI, median (IQR)^b^	26.3 (21.9–31.6)
Charlson Comorbidity Index, median (IQR)	4 (2–6)
Comorbid conditions	
Diabetes mellitus	59 (39.1)
Heart failure	43 (28.5)
Chronic kidney disease	39 (25.8)
Peripheral vascular disease	24 (15.9)
Chronic obstructive pulmonary disease	23 (15.2)
COVID-19	23 (15.2)
Chronic dialysis	18 (11.9)
Cystic fibrosis	18 (11.9)
Multidrug-resistant risk factors	
Antimicrobials for ≥24 h within prior 90 d	112 (74.2)
Hospitalization for ≥48 h within prior 90 d	108 (71.5)
Prior infection with resistant organisms	63 (41.7)
Colonization with resistant organisms	44 (29.1)
Admitted from nursing facility	30 (19.9)
Surgery within prior 30 d	22 (14.6)
Home infusion	17 (11.3)
Home wound care	10 (6.6)
Any immunosuppression factor^c^	29 (19.2)
Solid organ transplant within prior 90 d	11 (7.3)
Cytotoxic chemotherapy within prior 90 d	8 (5.3)
High-dose corticosteroids^d^	8 (5.3)
Neutropenia^e^	3 (2.0)
Functional or surgical asplenia	2 (1.3)
Bone marrow transplant within prior 90 d	1 (0.7)

Abbreviations: BMI, body mass index; COVID-19, coronavirus disease 2019; IQR, interquartile range.

^a^Data represent no. (%) of patients, unless otherwise specified.

^b^BMI calculated as weight in kilograms divided by height in meters squared.

^c^Four patients had >1 immunosuppression factor present.

^d^Defined as receipt of >200 mg hydrocortisone or equivalent for ≥2 weeks.

^e^Defined as absolute neutrophil count or white blood cell count <500/μL.

Infection characteristics are displayed in Table 2. The most common source of infection was the lower respiratory tract, accounting for 52.3% of cases. Bacteremia occurred in 19.2% of patients. Most patients (72.2%) received IMI/REL for targeted treatment of *P aeruginosa*. Other notable targeted pathogens included Enterobacterales (n = 49), *Enterococcus faecalis* (n = 8), *Mycobacterium abscessus* (n = 5), *Burkholderia* spp (n = 2), and *Acinetobacter baumannii* (n = 1). A polymicrobial index culture was present in 47.7%. Most patients had a carbapenem-nonsusceptible pathogen that was targeted by IMI/REL (85.4%). MDR *P aeruginosa* was present in 45.0% of patients, and 14.6% were infected with a DTR *P aeruginosa* isolate. CRE was present in 21.9%. At the time of index culture collection, most patients had a moderate- to high-risk Acute Physiology and Chronic Health Evaluation (APACHE) II score (median [IQR], 15 [10–23]); 43.7% were admitted to an intensive care unit (ICU). Of note, 7 patients did not have an index culture and IMI/REL was used empirically in these patients based on microbiologic history and/or clinical status.

**Table 2.** Infection Characteristics

Characteristic	Patients, No. (%)^a^ (n = 151)
Source of infection	
Lower respiratory tract	79 (52.3)
Skin/soft tissue	16 (10.6)
Urinary tract	14 (9.3)
Intra-abdominal, nonbiliary	11 (7.3)
Invasive prosthetic device	10 (6.6)
Intra-abdominal, biliary	7 (4.6)
Bone/joint	7 (4.6)
Other	4 (2.6)
Unknown	2 (1.3)
Intravenous catheter	1 (0.7)
Positive blood culture	29 (19.2)
Targeted pathogens	
*Pseudomonas aeruginosa*	109 (72.2)
*Klebsiella pneumoniae*	19 (12.6)
*Enterobacter cloacae* complex	8 (5.3)
*Enterococcus faecalis*	8 (5.3)
*Mycobacterium abscessus*	5 (3.3)
*Escherichia coli*	5 (3.3)
*Serratia marcescens*	4 (2.6)
*Klebsiella oxytoca*	3 (2.0)
*Proteus mirabilis*	3 (2.0)
*Providencia stuartii*	2 (1.3)
*Streptococcus* spp	2 (1.3)
*Citrobacter koseri*	2 (1.3)
*Burkholderia* spp	2 (1.3)
Other *Enterococcus* spp	2 (1.3)
*Raoultella* spp	2 (1.3)
*Acinetobacter baumannii*	1 (0.7)
*Citrobacter freundii* complex	1 (0.7)
*Pandoraea* spp	1 (0.7)
Polymicrobial index culture	73 (47.7)
Resistance phenotypes	
Any carbapenem-nonsusceptible isolate^b^	129 (85.4)
Carbapenem-resistant Enterobacterales spp^c^	33 (21.9)
MDR *P aeruginosa*^d^	68 (45.0)
DTR *P aeruginosa*^e^	22 (14.6)
Illness severity	
APACHE II score at index culture, median (IQR)^f^	15 (10–23)
ICU admission at time of index culture	66 (43.7)
Index culture specimen	
Sputum	40 (26.5)
Blood	24 (15.9)
Bronchoalveolar lavage	18 (11.9)
Wound	16 (10.6)
Body fluid	16 (10.6)
Tracheal aspirate	14 (9.3)
Tissue	11 (7.3)
Urine	10 (6.6)
Bone	4 (2.6)
Other	6 (4.0)
No index culture specimen	7 (4.6)

Abbreviations: APACHE II, Acute Physiology and Chronic Health Evaluation II; DTR, difficult-to-treat resistant; ICU, intensive care unit; IQR, interquartile range, MDR, multidrug resistant.

^a^Data represent no. (%) of patients, unless otherwise specified.

^b^Determined using Clinical and Laboratory Standards Institute M100 interpretive breakpoints.

^c^Defined as resistant to ≥1 carbapenem or known production of a carbapenemase.

^d^Defined as nonsusceptible to ≥3 antibiotic drug classes, excluding isolates meeting the DTR criteria below.

^e^Defined as nonsusceptible to all of the following: cefepime, ceftazidime, piperacillin-tazobactam, aztreonam, meropenem, imipenem-cilastatin, levofloxacin, and ciprofloxacin.

^f^Calculated using the worst physiologic parameters within 24 hours of index culture collection.

With regard to infection management (Table 3), most patients (94.7%) received an infectious diseases consultation. IMI/REL was initiated at a median (IQR) of 98.0 (49.0–178.0) hours after index culture collection and continued for a median (IQR) duration of 8.1 (6.0–13.8) days. IMI/REL was administered as directed therapy in 81.4% patients. Most patients received an appropriate dose, but 11.9% were underdosed per package insert recommendations based on their estimated CrCl calculated on the day of IMI/REL initiation. Concomitant systemic antibiotic therapy was administered to 24.5% of patients, and 23.2% received concomitant inhaled antibiotics. IMI/REL was most commonly selected due to a lack of active treatment options (48.3%), but it was also selected for regimen consolidation (17.2%) or for double coverage of a carbapenem-resistant organism (14.6%), among other reasons. Of note, 23 patients were continued on IMI/REL therapy following hospital discharge as an outpatient or while at a rehabilitation or nursing facility.

**Table 3.** Imipenem-Cilastatin-Relebactam Prescribing and Infection Management

Prescription and Management	Patients, No. (%)^a^ (n = 151)
IMI/REL dose^b^	
1250 mg	57 (37.7)
1000 mg	29 (19.2)
750 mg	42 (27.8)
500 mg	23 (15.2)
Frequency	
Every 6 h	149 (98.7)
Every 8 h	2 (1.3)
Acute kidney injury at IMI/REL initiation^c^	56 (37.1)
IMI/REL underdosed^d^	18 (11.9)
Active therapy before IMI/REL	38 (25.2)
IMI/REL directed therapy^e^	128 (84.7)
Concomitant systemic therapy for ≥24 h^f^	37 (24.5)
Aminoglycoside	10 (6.6)
Cefiderocol	6 (4.0)
Fluoroquinolone	5 (3.3)
Polymyxin B	4 (2.6)
Eravacycline	4 (2.6)
Aztreonam	3 (2.0)
Sulfamethoxazole-trimethoprim	3 (2.0)
Bedaquiline	1 (0.7)
Cefuroxime	1 (0.7)
Clofazimine	1 (0.7)
Colistin	1 (0.7)
Minocycline	1 (0.7)
Omadacycline	1 (0.7)
Tigecycline	1 (0.7)
Concomitant inhaled antibiotics^g^	35 (23.2)
Tobramycin	19 (12.6)
Colistin	15 (9.9)
Amikacin	5 (3.3)
Aztreonam	1 (0.7)
ID service consultation	143 (94.7)
Surgical consultation	45 (29.8)
Source control procedure performed	48 (31.8)
Ineligible for source control procedure^h^	79 (52.3)
Time to IMI/REL initiation, median (IQR), h^i^	98.0 (49.0–178.0)
Duration of IMI/REL therapy, median (IQR), d	8.1 (6.0–13.8)
Reason for IMI/REL selection^j^	
Lack of active treatment options	73 (48.3)
Regimen consolidation	26 (17.2)
Double coverage for carbapenem-resistant organism	22 (14.6)
Antibiotic shortage	15 (9.9)
History of MDR organism	15 (9.9)
Failure or worsening with prior therapy	7 (4.6)
Allergy or intolerance to alternative agents	3 (2.0)
Unknown	2 (1.3)
Lack of oral access	1 (0.7)
Other	7 (4.6)
Discharge disposition	
Home	56 (57.1)
Nursing facility^k^	42 (27.8)
Death	26 (27.2)
Rehabilitation center	11 (7.3)
Hospice	9 (6.0)
Other or unknown	7 (4.6)

Abbreviations: ID, infectious diseases; IMI/REL, imipenem-cilastatin-relebactam; IQR, interquartile range; MDR, multidrug resistant.

^a^Data represent no. (%) of patients, unless otherwise specified.

^b^Dose includes all components (imipenem, cilastatin, and relebactam), formulated in a 2:2:1 ratio.

^c^Acute kidney injury defined as an increase in serum creatinine by ≥0.3 mg/dL or a ≥50% increase from baseline or a new hemodialysis requirement on the day of IMI/REL initiation.

^d^Underdosing defined as administration of IMI/REL at a dose and/or frequency less than recommended according to the patient's creatinine clearance on the day of treatment initiation, as specified in the manufacturer's package insert.

^e^Receipt of IMI/REL for >24 hours following culture finalization.

^f^Patients could receive >1 concomitant systemic agent.

^g^Patients could receive >1 concomitant inhaled agent.

^h^Patients were presumed to be ineligible for a source control procedure if the infection originated from a respiratory or urinary source without a clear nidus of infection (abscess, empyema, or presence of prosthetic material) or if the source of infection was unknown.

^i^Calculated from index culture collection; patients without index culture or *Mycobacterium* spp infection were excluded due to the chronicity of this infection type.

^j^The total is >151 as a patient could have >1 reason for IMI/REL selection.

^k^Nursing home, skilled nursing facility, or long-term acute care.

Clinical outcomes are described in Table 4 for the overall cohort and in Supplementary Figure 1 for organisms of interest. Clinical success occurred in 70.2% of patients. The 30-day all-cause mortality rate was 18.5%. Microbiologic failure occurred in 14.6%; 11.9% experienced infection recurrence. An adverse drug effect occurred in 9 patients (6.0%). IMI/REL was discontinued in 3 of them due to nephrotoxicity (n = 1), neutropenia (n = 1), and a rash/dermatologic reaction (n = 1).

**Table 4.** Efficacy and Safety Outcomes

Outcome	Patients, No. (%)^a^ (n = 151)
Clinical success^b^	106 (70.2)
Secondary outcomes	
All-cause mortality within 30 d	28 (18.5)
Microbiologic failure within 30 d	22 (14.6)
Infection recurrence within 30 d	18 (11.9)
Length of hospital stay, median (IQR), d	25.0 (13.5–62.5)
Infection-related readmission within 30 d	5 (3.3)
Adverse drug reactions^c^	9 (6.0)
Gastrointestinal (nausea, vomiting, diarrhea)	3 (2.0)
Hepatoxicity	2 (1.3)
Nephrotoxicity	1 (0.7)
Encephalopathy	1 (0.7)
Neutropenia	1 (0.7)
Rash/dermatologic reaction	1 (0.7)
IMI/REL discontinued due to adverse drug reaction^d^	3 (2.0)

Abbreviations: IMI/REL, imipenem-cilastatin-relebactam; IQR, interquartile range.

^a^Data represent no. (%) of patients, unless otherwise specified.

^b^Clinical success was defined as improvement or resolution of infectious signs or symptoms during receipt of IMI/REL without 30-day microbiologic failure.

^c^Adverse drug reactions must be documented in the electronic medical record as associated with IMI/REL. Nephrotoxicity was defined as serum creatinine increase of ≥0.5 mg/dL and ≥50% from baseline on ≥2 occasions; hepatotoxicity, as elevations in aspartate aminotransferase or alanine aminotransferase levels; and neutropenia, as absolute neutrophil count (ANC) decrease to <1500/μL or 50% decrease if the baseline ANC was <1500/μL.

^d^IMI/REL was discontinued due to nephrotoxicity, neutropenia, or a rash/dermatologic reaction (each n = 1).

Variables selected for the multivariable logistic regression model (Supplementary Table 1) included heart failure, antimicrobials for ≥24 hours within the previous 90 days, polymicrobial index culture, DTR *P aeruginosa*, ICU admission at the time of index culture collection, and concomitant systemic antibiotic therapy ≥24 hours. Other variables with *P* values <.1 were not considered for model entry to avoid model overfitting given their low frequency within the dataset. The final model identified heart failure (adjusted odds ratio, 0.285 [95% confidence interval, .104–.785]), antimicrobials for ≥24 hours within the previous 90 days (0.161 [.033–.795]), DTR *P aeruginosa* (0.267 [.087–.817]), and ICU admission at the time of index culture collection (0.295 [.111–.786]) as independently associated with reduced odds of clinical success. The Hosmer-Lemeshow test demonstrated acceptable fit (*P* = .98).

**Figure 1.**
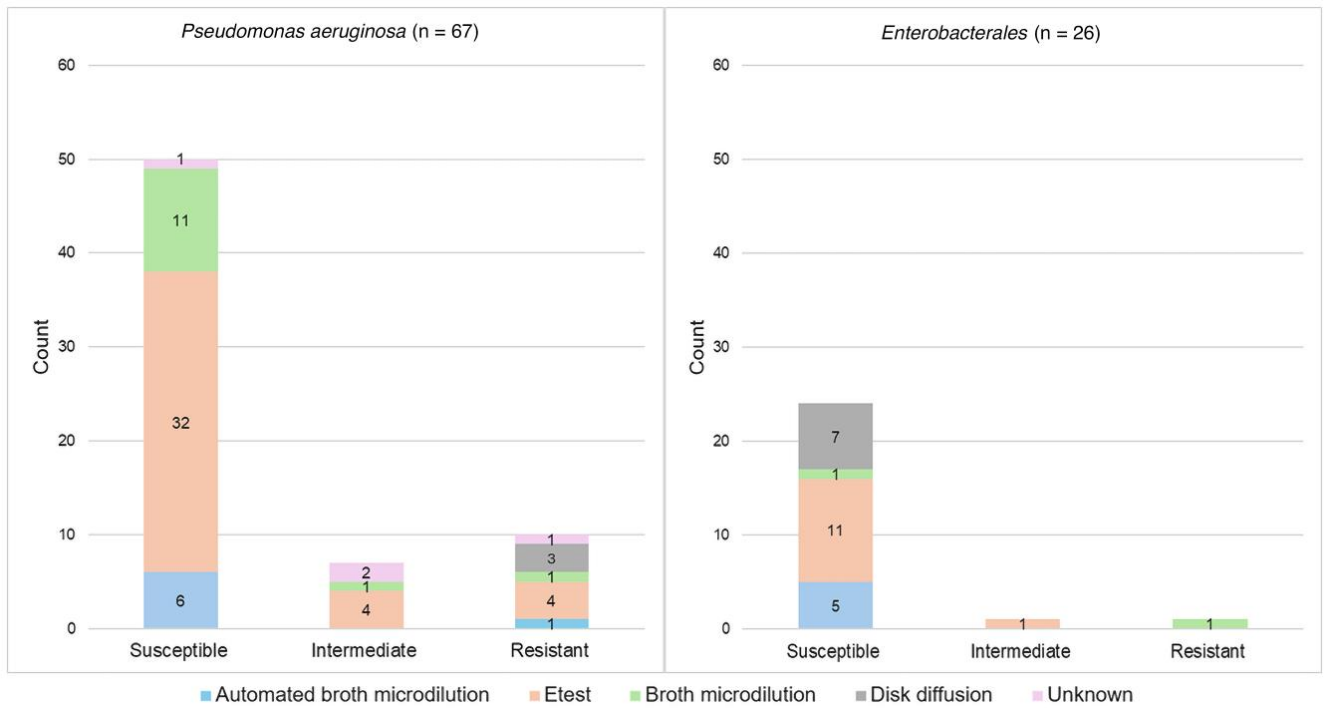
Imipenem-cilastatin-relebactam susceptibility testing methods and interpretation for *Pseudomonas aeruginosa* (n = 67) and Enterobacterales (n = 26) isolates.

Index culture finalization occurred at a median (IQR) of 6.0 (4.0–7.0) days from collection. IMI/REL susceptibility results were available for 96 isolates, including *P aeruginosa* (n = 67), Enterobacterales (n = 26), *Burkholderia* spp (n = 2) and *Pandoraea* spp (n = 1). A summary of susceptibility interpretation and testing methods for *P aeruginosa* and Enterobacterales is provided in Figure 1. Overall, most tested isolates were considered susceptible; however, 10 (14.9%) *P aeruginosa* isolates were resistant, and 7 (10.4%) demonstrated intermediate susceptibility to IMI/REL. Of the 17 patients with intermediately susceptible or resistant *P aeruginosa* isolates, 7 were changed to an alternative therapy. Ten patients remained on IMI/REL, 2 of whom experienced clinical failure. In addition, 1 patient each had intermediately susceptible and resistant Enterobacterales isolates, both of whom remained on IMI/REL and experienced clinical success. Among all patients with follow-up cultures, including those outside the 30-day microbiologic failure window (n = 36), IMI/REL susceptibility was available for 8 isolates, all of which were *P aeruginosa*. Among these 8 isolates, 4 (50%) were found to be resistant to IMI/REL. Of the patients with these 4 resistant isolates, the isolate from the index culture was either susceptible (n = 1), or resistant (n = 1) or had unknown susceptibility (n = 2) to IMI/REL.

## DISCUSSION

Our results offer valuable insights into the demographics and clinical characteristics of the real-world populations prescribed IMI/REL. We observed that IMI/REL was primarily used to treat infections caused by carbapenem-nonsusceptible bacteria among patients with a substantial comorbidity burden who would have been excluded from RCTs, including those with cystic fibrosis and various forms of immunosuppression [15, 16]. Furthermore, most patients had prior exposure to antibiotics and healthcare environments, exhibited high acuity of illness at infection onset, and had a long hospital stay. Although IMI/REL was predominantly used to treat lower respiratory tract infections, it was also used off-label for a diverse array of infection types. Overall, this study highlights the challenging clinical scenarios in which IMI/REL is prescribed, which is expected given its activity against MDR pathogens.

Clinical success was achieved in the majority of cases, with a 30-day all-cause mortality rate comparable to those in prior studies examining carbapenem-nonsusceptible infections [24–29]. These positive outcomes occurred despite a predominant lower respiratory tract source and delayed initiation of IMI/REL with a low frequency of active therapy before its administration, which have previously been associated with poor outcomes [25, 30–32]. In particular, comorbid heart failure, receipt of antibiotics in the past 90 days, ICU admission at the time of index culture collection, and isolation of DTR *P aeruginosa* were found to be independently associated with a reduced likelihood of clinical success. These findings illustrate the need for targeted investigations into management strategies for critically ill patients and/or those with a recent history of antibiotic exposure. Moreover, they corroborate previous findings suggesting that the presence of a DTR profile is predictive of poor clinical outcomes [22]. Overall, IMI/REL was also found to be safe and tolerable, with a low incidence of adverse drug events. Only 3 patients required drug discontinuation due to an adverse drug event, despite the use of this medication in a complex population. No novel adverse effects associated with IMI/REL were detected, as all adverse effects reported herein have previously been described in clinical trials and/or postmarketing surveillance [23].

IMI/REL was often used as targeted therapy to treat *P aeruginosa* infections, most of which were MDR or DTR. Notably, a polymicrobial index culture was present in almost half of patients, and IMI/REL was used for regimen consolidation in 17.2% of cases. These findings suggest that IMI/REL's ability to provide simultaneous coverage against MDR *P aeruginosa* and MDR Enterobacterales could have also contributed to preferential use in patients infected with multiple MDR gram-negative pathogens. IMI/REL offers a therapeutic advantage as it can overcome multiple carbapenem resistance mechanisms, including those specific to *P aeruginosa*, such as AmpC production, efflux pump, and porin channel mutations [11, 33]. Furthermore, it has demonstrated in vitro activity against *P aeruginosa* isolates that are resistant to ceftolozane-tazobactam and ceftazidime-avibactam, and it may be a viable salvage therapy in this situation [34, 35].

Among *P aeruginosa* isolates with susceptibility information, 10.4% (n = 7) displayed intermediate susceptibility and 14.9% (n = 10) were found to be resistant to IMl/REL. This rate of resistance is similar to that reported in epidemiologic studies and is likely reflective of patient complexity as well as healthcare and antibiotic exposure [12, 36, 37]. Notably, however, Etest was used for microbiologic susceptibility testing in some *P aeruginosa* isolates identified as either intermediate (n = 4) or resistant to IMI/REL (n = 4). The US Food and Drug Administration issued a class 2 device recall on Etest strips produced by bioMérieux in 2021 due to overcalling MICs among *P aeruginosa* isolates [38]. Thus, it is possible that this product was used to test isolates in this study and the number of intermediate and/or resistant isolates was falsely inflated.

Prior case series have also demonstrated IMI/REL to be efficacious and well tolerated in real-world settings, particularly among patients with MDR infections, including *P aeruginosa* and *K pneumoniae* carbapenemase–producing Enterobacterales [39–41]. Furthermore, a real-world study of 160 patients identified from an administrative database similarly found that IMI/REL was frequently prescribed among critically ill patients with comorbid conditions who were exposed to multiple antibiotics before its initiation. Although microbiologic data were limited to a subgroup of patients, MDR *P aeruginosa* was also the most frequently isolated organism in this study [40].

Our study has several limitations. First, as a retrospective, observational study without a comparator arm, it does not allow for definitive conclusions regarding the safety and efficacy of IMI/REL compared to other therapies. In addition, susceptibility testing was not universally performed on isolates. Follow-up cultures were not obtained for most patients, which restricts our ability to assess the development of resistance following IMI/REL exposure. In addition, data on renal function are available only for the first day of IMI/REL therapy in the included cohort, limiting our ability to assess the duration of potential underdosing and its impact on patient outcomes. Finally, the use of IMI/REL across various types of infections further complicates the ability to draw conclusions about its efficacy for specific indications and pathogens.

In summary, IMI/REL exhibited favorable efficacy and safety profiles when used to treat a complex patient demographic primarily facing carbapenem-nonsusceptible infections, in which treatment options are often limited. Our findings highlight the potential of IMI/REL as a valuable therapeutic option in challenging clinical scenarios. Continued investigation into its application among specific situations, including comparative analyses, will be important to more completely determine its place in therapy to combat the escalating threat of MDR gram-negative infections.

## Supplementary Material

ofaf112_Supplementary_Data

## Supplementary Data

Supplementary materials are available at *Open Forum Infectious Diseases* online. Consisting of data provided by the authors to benefit the reader, the posted materials are not copyedited and are the sole responsibility of the authors, so questions or comments should be addressed to the corresponding author.
