# Supplementary material for: Real-World Applications of Imipenem-Cilastatin-Relebactam: Insights From a Multicenter Observational Cohort Study
Source: Open Forum Infect Dis. 2025 Feb 26;12(4):ofaf112. doi: 10.1093/ofid/ofaf112 (PMC11983095; doi:10.1093/ofid/ofaf112)
Supplement: ofaf112_Supplementary_Data [file ofaf112_supplementary_data.docx]

| **Supplementary Figure 1.** Clinical efficacy outcome overall and by pathogen of interest. |
| --- |


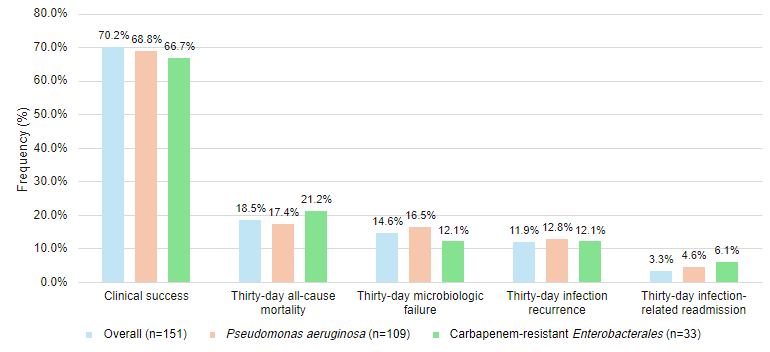


| **Supplementary Table 1.** Clinical success vs. no clinical success.^1^ | | | |
| --- | --- | --- | --- |
|  | **No clinical success**  **(n = 45)** | **Clinical success**  **(n = 106)** | **p-value** |
| Age, years | 54 (40.5-69.5) | 60.0 (43.5-72.0) | 0.249 |
| Sex, male | 24 (53.3) | 58 (54.7) | 0.876 |
| Race |  |  |  |
| Non-Hispanic Caucasian | 27 (60.0) | 76 (71.7) | 0.158 |
| Black/African American | 9 (20.0) | 24 (22.6) | 0.719 |
| Hispanic | 3 (6.7) | 5 (4.7) | 0.696 |
| Asian | 5 (11.1) | 0 | 0.002 |
| Other/unknown | 1 (2.2) | 1 (0.9) | 0.509 |
| BMI, kg/m^2^ | 26.0 (22.3-32.5) | 26.4 (21.6-30.3) | 0.782 |
| Charlson comorbidity index | 4 (1-5) | 4 (2-6) | 0.290 |
| Comorbid conditions |  |  |  |
| Diabetes mellitus | 17 (37.8) | 42 (39.6) | 0.832 |
| Heart failure | 17 (37.8) | 26 (24.5) | 0.099 |
| Chronic kidney disease | 14 (31.1) | 25 (23.6) | 0.334 |
| Peripheral vascular disease | 6 (13.3) | 18 (17.0) | 0.575 |
| Chronic obstructive pulmonary disease | 4 (8.9) | 19 (17.9) | 0.217 |
| COVID-19 | 10 (22.2) | 13 (12.3) | 0.119 |
| Chronic dialysis | 7 (15.6) | 11 (10.4) | 0.369 |
| Cystic fibrosis | 7 (15.6) | 11 (10.4) | 0.369 |
| Acute kidney injury at IMI/REL initiation^2^ | 20 (44.4) | 36 (34.0) | 0.223 |
| Multidrug-resistant risk factors |  |  |  |
| Antimicrobials ≥24 hours in previous 90 days | 40 (88.9) | 72 (67.9) | 0.007 |
| Hospitalization ≥48 hours in previous 90 days | 35 (32.4) | 73 (67.6) | 0.267 |
| Prior infection with resistant organisms | 23 (51.1) | 40 (63.5) | 0.127 |
| Colonization with resistant organisms | 15 (33.3) | 29 (27.4) | 0.460 |
| Admitted from nursing facility | 6 (13.3) | 24 (22.6) | 0.190 |
| Surgery in previous 30 days | 4 (8.9) | 18 (17.0) | 0.312 |
| Home infusion | 5 (11.1) | 12 (11.3) | 0.970 |
| Home wound care | 2 (4.4) | 8 (7.5) | 0.724 |
| Any immunosuppression factor^3^ | 7 (15.6) | 22 (20.8) | 0.458 |
| Neutropenia^4^ | 1 (2.2) | 2 (1.9) | 1.000 |
| Functional or surgical asplenia | 1 (2.2) | 1 (0.9) | 0.509 |
| Solid organ transplant in previous 90 days | 3 (6.7) | 8 (7.5) | 1.000 |
| Cytotoxic chemotherapy in previous 90 days | 0 | 8 (7.5) | 0.106 |
| High-dose corticosteroids^5^ | 2 (4.4) | 6 (5.7) | 1.000 |
| Bone marrow transplant in previous 90 days | 0 | 1 (0.9) | 1.000 |

|  | **No clinical success**  **(n = 45)** | **Clinical success**  **(n = 106)** | **p-value** |
| --- | --- | --- | --- |
| Source of infection |  |  |  |
| Lower respiratory tract | 28 (62.2) | 51 (48.1) | 0.112 |
| Skin/soft tissue | 4 (8.9) | 12 (11.3) | 0.778 |
| Urinary tract | 2 (4.4) | 12 (11.3) | 0.232 |
| Intra-abdominal, non-biliary | 0 | 11 (10.4) | 0.034 |
| Invasive prosthetic device | 7 (15.6) | 3 (2.8) | 0.008 |
| Intra-abdominal, biliary | 0 | 7 (6.6) | 0.104 |
| Bone/joint | 2 (4.4) | 5 (4.7) | 1.000 |
| Other | 1 (2.2) | 3 (2.8) | 1.000 |
| Unknown | 0 | 2 (1.9) | 0.354 |
| Intravenous catheter | 1 (2.2) | 0 | 0.298 |
| Positive blood culture | 11 (24.4) | 18 (17.0) | 0.287 |
| Polymicrobial index culture | 15 (33.3) | 57 (53.8) | 0.021 |
| Resistance phenotypes |  |  |  |
| Carbapenem non-susceptible^6^ | 41 (91.1) | 88 (83.0) | 0.312 |
| Carbapenem-resistant *Enterobacterales*^7^ | 11 (24.4) | 22 (20.8) | 0.616 |
| MDR *P. aeruginosa*^8^ | 19 (42.2) | 49 (46.2) | 0.722 |
| DTR *P. aeruginosa*^9^ | 13 (38.2) | 9 (12.0) | 0.002 |
| IMI/REL susceptibility available | 26 (57.8) | 70 (66.0) | 0.335 |
| IMI/REL resistance | 4 (8.9) | 8 (7.5) | 0.751 |
| Illness severity |  |  |  |
| APACHE II score at index culture^10^ | 14 (8-24) | 15 (10-22) | 0.863 |
| Intensive care unit at time of index culture | 30 (69.8) | 36 (35.3) | <0.001 |
| Management |  |  |  |
| IMI/REL underdosed^11^ | 7 (15.6) | 11 (10.4) | 0.369 |
| Active therapy prior to IMI/REL | 11 (25.6) | 27 (26.5) | 0.894 |
| IMI/REL directed therapy^12^ | 35 (77.8) | 88 (83.0) | 0.448 |
| Concomitant systemic antibiotic therapy ≥24 hours | 16 (35.6) | 21 (19.8) | 0.040 |
| Concomitant inhaled antibiotics | 13 (28.9) | 22 (20.8) | 0.279 |
| Infectious diseases service consult | 42 (93.3) | 101 (95.3) | 0.696 |
| Surgical consult | 12 (26.7) | 33 (31.7) | 0.536 |
| Source control procedure | 15 (33.3) | 33 (31.1) | 0.790 |
| IMI/REL initiation <48 hours of index culture | 11 (24.4) | 25 (23.6) | 0.910 |
| 1. Data presented as median (interquartile range [IQR]) or n (%), as appropriate. 2. Defined as an increase in serum creatinine by ≥0.3 mg/dL or ≥1.5 times increase from baseline or new hemodialysis requirement on day of imipenem/cilastatin/relebactam initiation. 3. Patients could have >1 immunosuppression factor present. 4. Defined as absolute neutrophil count or white blood cell count <500 cells/mL. 5. Defined as receipt of >200 mg hydrocortisone or equivalent for ≥2 weeks. 6. Determined utilizing Clinical and Laboratory Institute (CLSI) M100 interpretive breakpoints. 7. Defined as resistant to ≥1 carbapenem or known possession of a carbapenemase. 8. Defined as non-susceptibility to ≥3 antibiotic drug classes; excluding isolates meeting the difficult-to-treat criteria below. 9. Defined as non-susceptible to all of the following: cefepime, ceftazidime, piperacillin/tazobactam, aztreonam, meropenem, imipenem/cilastatin, levofloxacin, and ciprofloxacin. 10. Calculated using the worst physiologic parameters within 24 hours of index culture attainment. 11. Defined as administration of IMI/REL at a dose and/or frequency less than the recommended dose according to the patient’s creatinine clearance on the day of treatment initiation, as specified in the manufacturer’s package insert. 12. Receipt of IMI/REL for >24 hours following culture finalization.   Abbreviations: BMI, body mass index; IMI/REL, imipenem/cilastatin/relebactam; MDR, multidrug-resistant; DTR, difficult-to-treat; APACHE II, acute physiology and chronic health evaluation II | | | |
